# Proteomic analysis of cardiorespiratory fitness for prediction of mortality and multisystem disease risks

**DOI:** 10.1038/s41591-024-03039-x

**Published:** 2024-06-04

**Authors:** Andrew S. Perry, Eric Farber-Eger, Tomas Gonzales, Toshiko Tanaka, Jeremy M. Robbins, Venkatesh L. Murthy, Lindsey K. Stolze, Shilin Zhao, Shi Huang, Laura A. Colangelo, Shuliang Deng, Lifang Hou, Donald M. Lloyd-Jones, Keenan A. Walker, Luigi Ferrucci, Eleanor L. Watts, Jacob L. Barber, Prashant Rao, Michael Y. Mi, Kelley Pettee Gabriel, Bjoern Hornikel, Stephen Sidney, Nicholas Houstis, Gregory D. Lewis, Gabrielle Y. Liu, Bharat Thyagarajan, Sadiya S. Khan, Bina Choi, George Washko, Ravi Kalhan, Nick Wareham, Claude Bouchard, Mark A. Sarzynski, Robert E. Gerszten, Soren Brage, Quinn S. Wells, Matthew Nayor, Ravi V. Shah

**Affiliations:** 1 Vanderbilt Translational and Clinical Cardiovascular Research Center, Vanderbilt University School of Medicine, Nashville, TN USA; 2 MRC Epidemiology Unit, University of Cambridge, Cambridge, UK; 3 Longtidudinal Studies Section, Translational Gerontology Branch, National Institute on Aging, NIH, Baltimore, MD USA; 4 Cardiovascular Institute, Beth Israel Deaconess Medical Center, Harvard Medical School, Boston, MA USA; 5 Department of Medicine, University of Michigan, Ann Arbor, MI USA; 6 Department of Biostatistics, Vanderbilt University Medical Center, Nashville, TN USA; 7 Department of Preventive Medicine, Northwestern University Feinberg School of Medicine, Chicago, IL USA; 8 Multimodal Imaging of Neurodegenerative Disease (MIND) Unit, National Institute on Aging, NIH, Baltimore, MD USA; 9 Division of Cancer Epidemiology and Genetics, National Cancer Institute, Rockville, MD USA; 10 Department of Epidemiology, University of Alabama at Birmingham, Birmingham, AL USA; 11 Kaiser Permanente, Oakland, CA USA; 12 Cardiology Division, Massachusetts General Hospital, Boston, MA USA; 13 Division of Pulmonary, Critical Care, and Sleep Medicine, Department of Medicine, University of California Davis, Sacramento, CA USA; 14 Department of Laboratory Medicine and Pathology, University of Minnesota, Minnesota, MN USA; 15 Department of Medicine, Northwestern University Feinberg School of Medicine, Chicago, IL USA; 16 Division of Pulmonary and Critical Care Medicine, Department of Medicine, Brigham and Women’s Hospital, Boston, MA USA; 17 Division of Pulmonary and Critical Care Medicine, Department of Medicine, Northwestern University Feinberg School of Medicine, Chicago, IL USA; 18 Human Genomic Laboratory, Pennington Biomedical Research Center, Baton Rouge, LA USA; 19 Department of Exercise Science, University of South Carolina Columbia, Columbia, SC USA; 20 Sections of Cardiovascular Medicine and Preventive Medicine and Epidemiology, Department of Medicine, Boston University School of Medicine, Boston, MA USA

**Keywords:** Prognostic markers, Epidemiology

## Abstract

Despite the wide effects of cardiorespiratory fitness (CRF) on metabolic, cardiovascular, pulmonary and neurological health, challenges in the feasibility and reproducibility of CRF measurements have impeded its use for clinical decision-making. Here we link proteomic profiles to CRF in 14,145 individuals across four international cohorts with diverse CRF ascertainment methods to establish, validate and characterize a proteomic CRF score. In a cohort of around 22,000 individuals in the UK Biobank, a proteomic CRF score was associated with a reduced risk of all-cause mortality (unadjusted hazard ratio 0.50 (95% confidence interval 0.48–0.52) per 1 s.d. increase). The proteomic CRF score was also associated with multisystem disease risk and provided risk reclassification and discrimination beyond clinical risk factors, as well as modulating high polygenic risk of certain diseases. Finally, we observed dynamicity of the proteomic CRF score in individuals who undertook a 20-week exercise training program and an association of the score with the degree of the effect of training on CRF, suggesting potential use of the score for personalization of exercise recommendations. These results indicate that population-based proteomics provides biologically relevant molecular readouts of CRF that are additive to genetic risk, potentially modifiable and clinically translatable.

## Abstract (web summary)

A proteomic risk score for cardiorespiratory fitness, comprising as few as 21 proteins, is dynamic with exercise training and helps predict the risk of mortality and a range of cardiovascular, metabolic and neurological conditions.

## Main

CRF is a powerful prognostic marker linked to greater health, quality of life and longevity across the life course^1–6^. Measuring CRF is an important component of clinical care in several disease conditions^3,7^ and is often considered an essential health metric on par with clinical vital signs^6^. Nevertheless, widespread clinical assessment of CRF for risk stratification and health promotion has been limited by test availability, cost and factors (for example, musculoskeletal) that may limit the ability to perform maximum effort exercise. An alternative approach—easily accessible, training-responsive biomarkers of CRF—may address these limitations and enable discovery of pharmacological targets that mimic effects of exercise. Exercise is accompanied by widespread changes in the human metabolic state, spanning pathways of tissue regeneration and fibrosis, muscle structure, mitochondrial dysfunction, insulin resistance and inflammation^8–12^. While molecular surrogates of CRF and training responses are associated with clinical prognosis^8,10,13^, most studies have been across a single population with limited follow-up and outcomes and have demonstrated effect sizes that are not significantly additive over standard risk factors.

Here, we performed an international population-based study of 14,145 individuals with CRF measures spanning four different population-based observational cohorts (the Coronary Artery Risk Development in Young Adults (CARDIA) study; the Fenland Study; the Baltimore Longitudinal Study of Aging (BLSA); and the Health, Risk Factors, Exercise Training and Genetics (HERITAGE) family sutdy) with diverse modes of CRF assessment to define and validate a proteomic signature of CRF. Leveraging data from around 22,000 participants from the UK Biobank (UKB), we tested the association of a proteomic signature of CRF with a broad array of clinical outcomes (death, cardiovascular, metabolic, malignancy, neurological) and examined the interaction with polygenic risk. In HERITAGE, we evaluated whether a 20-week exercise training program modified a proteomic signature of CRF. To our knowledge, this study provides the largest, most comprehensive human population-based proteomic study of CRF, demonstrating its broad functional and clinical relevance to human disease with a path for clinical translation.

## Results

### Characteristics of study samples

Our initial sample to establish relations of the circulating proteome with CRF included participants from CARDIA. The CARDIA sample consisted of 2,238 individuals with a median age 51 years (56% female, 43% Black; Table 1). CARDIA participants were generally overweight (median body mass index (BMI) 29 kg m^−2^) with a modest prevalence of diabetes (14%) and treated hypertension (26%). We did not observe any important differences between our CARDIA derivation (70%) and validation (30%) subsets (split randomly, balanced on exercise treadmill test (ETT) time). We validated our findings in three external cohorts: Fenland^14^; BLSA^15^; and HERITAGE^10^. These cohorts spanned early to older adulthood with a wide range of BMI and comorbidity (Supplementary Table 1a). A subsample of the UKB (*N* = 21,988; median age 58 years, 54% female, 93% white; Supplementary Table 1b) with available proteomics was used to test the association of the CRF proteome with a broad array of outcomes. The method of CRF assessment differed across cohorts (Methods), which—in conjunction with cohort-specific differences (for example, age)—contributed to differences in CRF distributions.

**Table 1:** Baseline characteristics of the CARDIA study population

Characteristic	Overall *n* = 2,238	Derivation *n* = 1,569	Validation *n* = 669	*P* value
Age (years)	51.0 (47.0, 53.0); 0%	50.0 (47.0, 53.0); 0%	51.0 (48.0, 54.0); 0%	0.015
Sex, *n* (%)				>0.9
Male	978 (44%); 0%	686 (44%); 0%	292 (44%); 0%	
Female	1,260 (56%); 0%	883 (56%); 0%	377 (56%); 0%	
Race, *n* (%)				0.3
Black	973 (43%); 0%	670 (43%); 0%	303 (45%); 0%	
White	1,265 (57%); 0%	899 (57%); 0%	366 (55%); 0%	
CARDIA Field Center, *n* (%)				0.7
Birmingham	531 (24%); 0%	362 (23%); 0%	169 (25%); 0%	
Chicago	564 (25%); 0%	403 (26%); 0%	161 (24%); 0%	
Minnesota	523 (23%); 0%	368 (23%); 0%	155 (23%); 0%	
Oakland	620 (28%); 0%	436 (28%); 0%	184 (28%); 0%	
Body mass index (kg m^−2^)	29 (25, 33); <0.1%	29 (25, 33); <0.1%	28 (25, 33); 0%	0.8
Lifetime smoking pack years	0 (0, 5); 0%	0 (0, 5); 0%	0 (0, 7); 0%	0.5
Systolic blood pressure (mmHg)	116 (108, 126); <0.1%	116 (107, 126); 0%	116 (108, 125); 0.1%	0.7
Diastolic blood pressure (mmHg)	73 (66, 80); <0.1%	73 (66, 80); 0%	72 (66, 80); 0.3%	0.8
Treated for hypertension, *n* (%)	583 (26%); 0%	395 (25%); 0%	188 (28%); 0%	0.15
Diabetes, *n* (%)	313 (14%); 0%	210 (13%); 0%	103 (15%); 0%	0.2
History of CVD	44 (2.0%); 0%	36 (2.3%); 0%	8 (1.2%); 0%	0.5
eGFR (ml min^−1^ 1.73m^−2^)	94 (82, 107); <0.1%	93 (82, 106); <0.1%	94 (83, 108); 0.1%	0.087
Total cholesterol (mg dl^−1^)	190 (167, 215); 0%	190 (167, 215); 0%	190 (166, 215); 0%	0.5
High density lipoprotein (mg dl^−1^)	55 (45, 67); 0%	56 (45, 67); 0%	54 (45, 67); 0%	0.7
Year 20 ETT time (s)	420 (304, 539); 0%	420 (304, 539); 0%	420 (304, 539); 0%	>0.9

The study population was split into derivation/validation samples, balanced by Year 20 ETT time. Continuous variables are reported at median (25th, 75th percentile) with percentage missingness. Categorical variables are reported as *n* (%) with percentage missingness. Reported *P* values are from two-sided Wilcoxon tests (for continuous variables) and two-sided Chi-square tests (categorical variables).

### Development of a proteomic CRF score

**Fig. 1:**
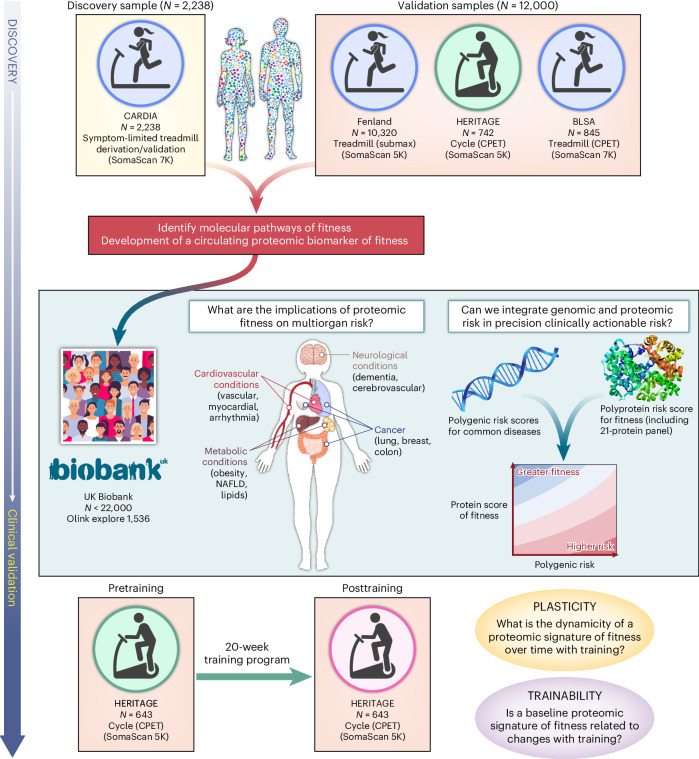
Study design. We developed and validated a circulating proteomic signature of CRF across four cohorts and various exercise modalities. In the UKB, we examined the relationship a proteomic CRF signature with a broad range of clinical endpoints and examined its interaction with polygenic risk. In HERITAGE, we examined the association of the proteomic CRF signature with response to exercise training and correlated changes in signature with changes in CRF. NAFLD, nonalcoholic fatty liver disease.

**Fig. 2:**
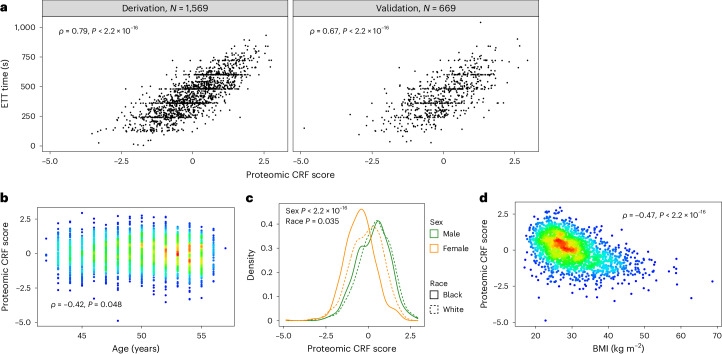
Development of the proteomic CRF score in CARDIA. **a**, Correlations between the proteomic CRF score and CRF (defined by ETT time) in CARDIA across derivation (left) and validation (right) samples. **b**–**d**, Correlations of the proteomic CRF score with age (**b**), sex and race (**c**) and BMI (**d**). Colors on scatter plots represent density of overlapping observations, with red being the most dense and blue the least dense. *P* values in **a**, **b** and **d** are from Spearman rank correlation tests. *P* values in **c** are from linear regression modeling of the proteomic CRF score as a function of sex and race. All *P* values are from two-sided tests.

We sought to develop an integrative score of CRF to leverage the multiorgan and diverse drivers of CRF. Using penalized regression (least absolute shrinkage and selection operator (LASSO)) across the assayed proteome, we developed a proteomic CRF score in the CARDIA derivation subset, using ETT time as the CRF measure, and validated it across approximately 12,500 participants across four samples (Fig. 1). We achieved a >95% reduction in proteomic space (272 aptamers selected from 7,230 candidates) with good calibration in both the CARDIA derivation (Spearmanʼs *ρ* = 0.79) and validation subsets (Spearmanʼs *ρ* = 0.67; Fig. 2), comparable with previously published metabolomic^13^ or proteomic instruments^16^. We observed mechanistically plausible directionality for many of the proteins of the highest effect sizes (Table 2), including proteins implicated in innate immunity and inflammation (C5a^17,18^), atherosclerosis (AGER^19^, RGMB^19^), neuronal survival and growth (CDNF^20^, LSAMP^21^), cell physiology (TNR—migration, adhesion, differentiation; DUSP13—differentiation, proliferation), oxidative stress (MRM1^22^), energy expenditure and substrate fuel utilization (OLFM2^23^, FABP4^24^, FABP3^25^, HNF4A^26^, GLYATL2), adiposity (LEP, CA6^27^), peripheral muscle responses to exercise (MB^28^, ATF6^29^) and autophagy (GLIPR2^30^).

**Table 2:** Biological curation of selected CRF-related proteins

Gene (protein)	LASSO directionality	Molecular evidence
*C5* (C5a anaphylatoxin)	−	Pro-inflammatory response to complement activation; rise with acute exercise; may have cross-tissue roles in innate immune activation, lipid metabolism and survival^17,18^
*CDNF* (cerebral dopamine neurotrophic factor)	+	Central nervous system expression, involved in neuronal survival^20^; Increases in spinal cord with exercise in Parkinsonism^67^
*GLIPR2* (Golgi-associated plant pathogenesis-related protein 1)	+	Negative regulator of autophagy^30^
*LEP* (leptin)	−	Adipocyte product, implicated in obesity pathogenesis; previous associations with fitness
*OLFM2* (noelin-2)	−	Deficiency is protective against diet-induced obesity via reduced energy intake and augmented energy expenditure owing to brown adipose tissue thermogenesis and fat browning^23^
*HTRA1* (serine protease HTRA1)	−	Serine protease; pleotropic effects on protein metabolism, signaling, skeletal muscle physiology and bone growth; deficiency leads to increased bone growth, potentially via modulation of TGFβ signaling^68^
*LSAMP* (limbic system-associated membrane protein)	−	Growth of neurons in limbic system^21^
*MB* (myoglobin)	+	Muscle product; increased during chronic exercise^28^
*ATF6* (cyclic AMP-dependent transcription factor ATF6 alpha)	+	Involved in unfolded protein response during ER stress; unfolded protein response activation in peripheral muscle during exercise is adaptive and facilitates recovery^29^
*EWSR1* (RNA-binding protein EWS)	−	Nucleic acid binding protein; involved in regulation of transcription and posttranscriptional events^69^
*PLXNA1* (plexin-A1)	−	Involved in semaphorin signaling
*FABP3* (fatty acid binding protein, heart)	−	Involved in lipid handling in skeletal and cardiac muscle; elevated levels in myocardial infarction (potentially from cellular release)^25^
*PDHA2* (pyruvate dehydrogenase E1 component subunit alpha, testis-specific form, mitochondrial)	−	Expressed in testis; unclear connection to fitness
*F10* (coagulation factor Xa)	+	Coagulation factor
*CA6* (carbonic anhydrase 6)	+	Also known as gustin; involved in taste perception; genetic studies reveal role in adiposity^27^
*NCBP1* (nuclear cap-binding protein subunit 1)	−	Involved in mRNA processing
*SVEP1* (Sushi, von Willebrand factor type A, EGF and pentraxin domain-containing protein 1)	−	Vascular smooth muscle cell product; implicated in atherosclerosis development^70^
*HNF4A* (hepatocyte nuclear factor 4-alpha)	−	Transcription factor; involved in regulation of lipid and carbohydrate metabolism in the liver, including gluconeogenesis^26^
*CRISP2* (cysteine-rich secretory protein 2)	+	Expressed in testis; unclear connection to fitness
*FABP4* (fatty acid binding protein, adipocyte)	−	Regulation of lipid metabolism; increased after acute exercise^24^; increased circulating FABP4 associated with insulin resistance^71^

The top 20 CRF-related proteins (LASSO regression) were examined via literature search to assess potential implications in metabolic disease and health.

**Extended Data Fig. 1:**
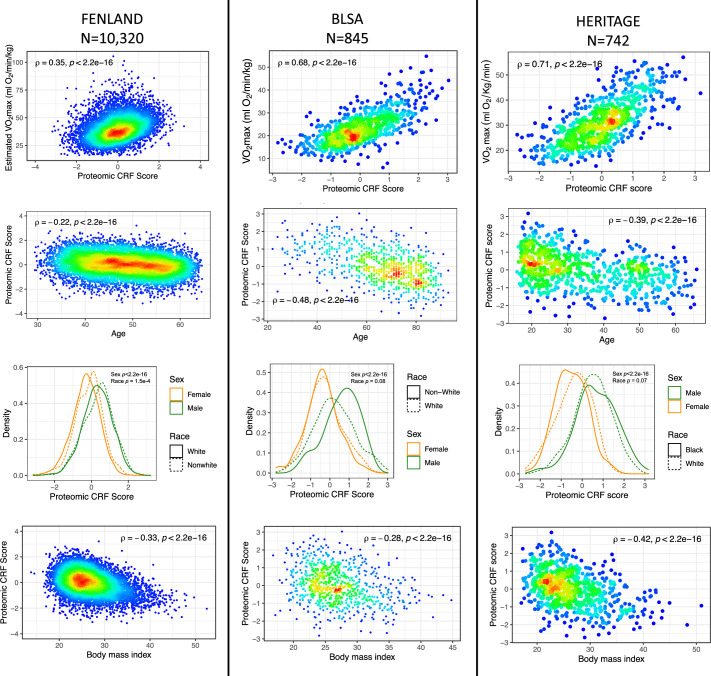
Relationship of a protein score of fitness with VO_2_ max, age, sex, race and BMI in 3 validation cohorts. The proteomic CRF score was scaled (mean 0, variance 1) in BLSA and HERITAGE cohorts. Colors on scatter plots represent density of overlapping observations with red being the most dense and blue the least dense. P values on panels showing the relationship of the proteomic CRF score with sex and race are from linear regression models of the proteomic CRF score as a function of sex and race. All other panels report P values from Spearman rank correlation tests. P values below 2.2 × 10^–16^ are reported as p < 2.2e-16.

**Extended Data Fig. 2:**
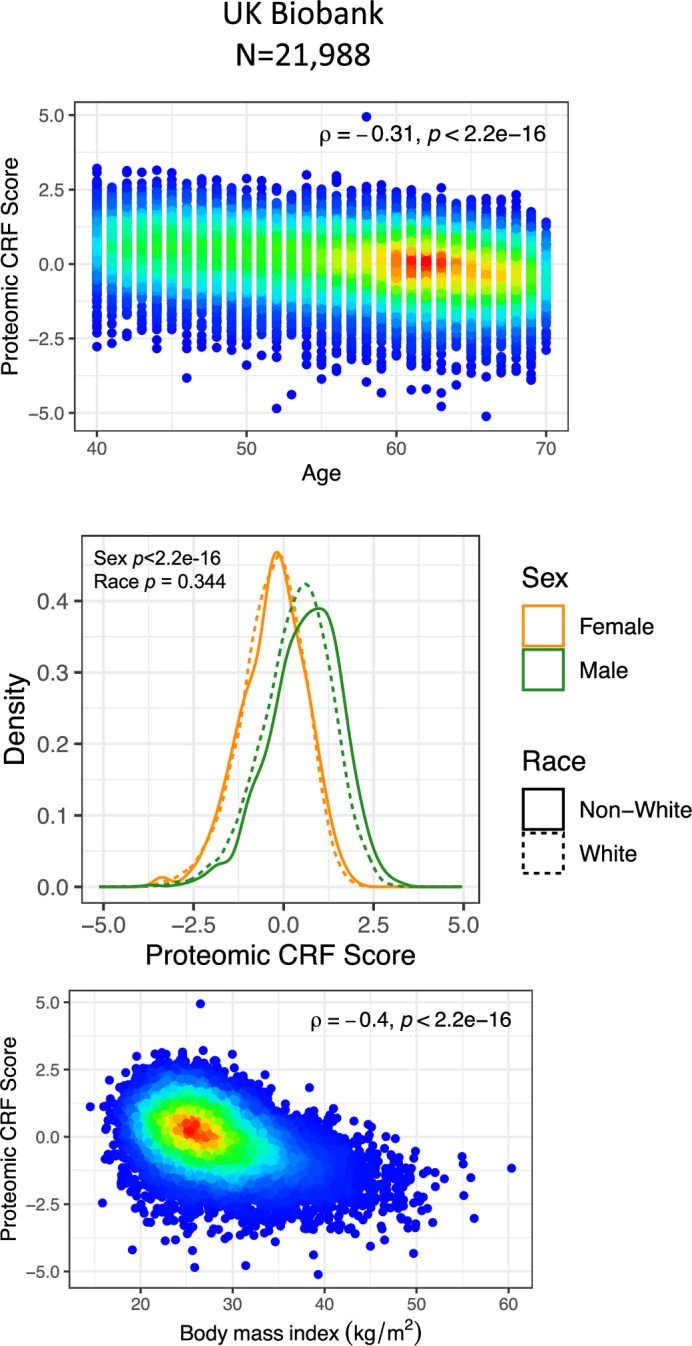
Relations of a protein score of fitness with age, sex, race and BMI in UK Biobank. Colors on scatter plots represent density of overlapping observations with red being the most dense and blue the least dense. P values on panels showing the relationship of the proteomic CRF score with sex and race are from linear regression models of the proteomic CRF score as a function of sex and race. All other panels report P values from Spearman rank correlation tests. P values below 2.2 × 10^–16^ are reported as p < 2.2e-16.

After recalibration to shared proteins across each of our validation samples (Fenland, HERITAGE, BLSA; Supplementary Tables 3–5 and Methods), we observed differences in fit against measured CRF, most likely owing to heterogeneity in methods for assessment of CRF (Extended Data Fig. 1). The best validation fits were observed in HERITAGE (*ρ* = 0.71) and BLSA (*ρ* = 0.68), where CRF was assessed by symptom-limited peak exercise testing with directly measured gas exchange (peak VO_2_). The weakest validation fit was observed in Fenland (*ρ* = 0.35), where CRF was estimated from heartrate response to submaximal exercise with extrapolation to age-predicted maximal heartrate. We observed consistent differences in the proteomic CRF score by sex (men higher) and inverse associations with age and BMI (Extended Data Figs. 1 and 2), consistent with the general epidemiology of CRF^14^.

### Relations of a proteomic CRF score with clinical outcomes

**Fig. 3:**
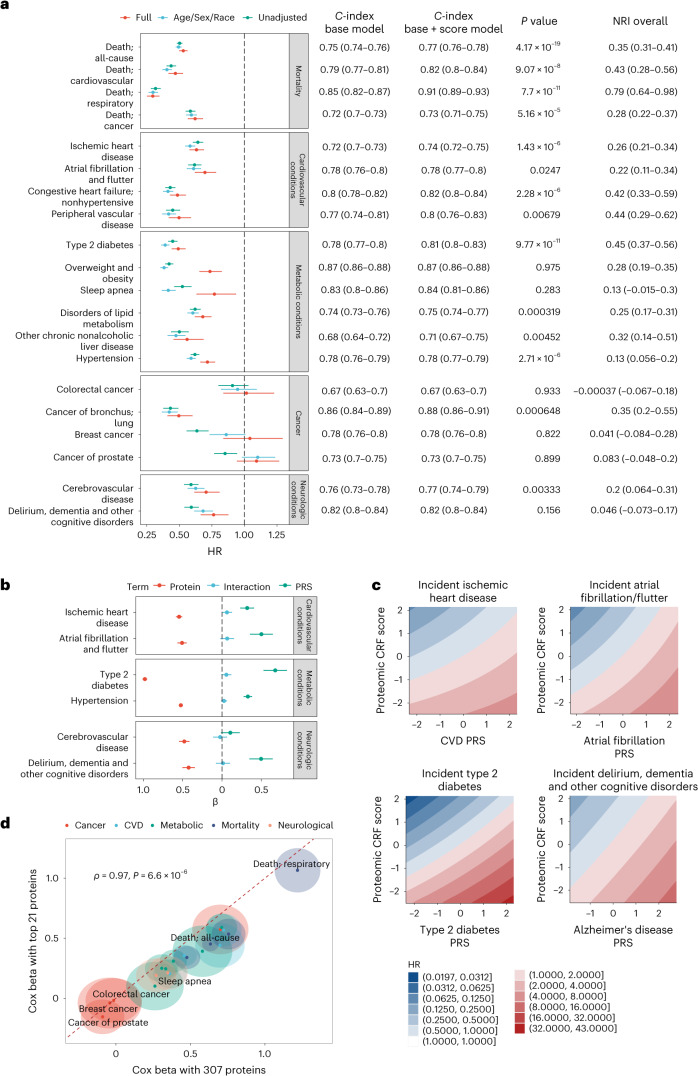
Proteomic CRF score, polygenic risk and multisystem clinical outcomes. **a**, Forest plot of Cox model results with proteomic score as the main predictor, grouped by outcome category. The ‘full’ adjustment model includes adjustment for age, sex, race, BMI, systolic blood pressure, diabetes, Townsend deprivation index, smoking, alcohol and LDL. Error bars, 95% CI. The adjoining table reports the *C*-index for Cox models without proteomic score (Base) and with the score (Score). Base models include age, sex, race, BMI, systolic blood pressure, diabetes, Townsend deprivation index, smoking, alcohol and LDL. Reported *P* value is from comparison testing of C-indices by *z* distribution (two-sided) without correct for multiple comparison. **b**, Cox beta coefficients from models including an interaction between the protein score of CRF and PRSs of the indicated conditions or diseases. Error bars, 95% CI. **c**, Contour map of the model predicted HR across the range of protein score of fitness and PRSs. The referent hazard was set at the median of the protein score and median of the PRS. Values reported and visualized are from point estimates and 95% CI. **d**, Comparison of Cox model coefficients from a parsimonious 21-protein panel and the full 307-protein panel. The halo represents the 95% CI around the model coefficient. *P* value is from two-sided Spearman rank correlation test. For visualization, we reversed the sign of the beta coefficients. Full data on sample sizes, model estimates and results of statistical testing may be found in Supplementary Tables 7 and 13.

Given the multicohort replication of the proteomic CRF score and its biological plausibility, we next sought to test its clinical relevance. We identified a sample of 21,988 UKB participants with proteomic data (Olink Explore 1536) and with survival data for a wide array of outcomes (Supplementary Table 1b). Over a median follow-up of 13.7 years (25th–75th percentile, 13.0–14.5 years), 2,394 deaths occurred (other outcomes reported in Supplementary Table 7). Per each 1 s.d. higher CRF proteome score, we observed a near 50% lower hazard of all-cause mortality (hazard ratio (HR) = 0.53, 95% confidence interval (CI) 0.50–0.56; *P* < 0.0001) and cause-specific mortality (Fig. 3a; all HRs and 95% CIs in Supplementary Table 7), robust to adjustment for standard clinical risk factors and bioimpedance-based measured fat mass. In addition to censoring at other causes of death for models for cause-specific mortality, we observed similar results using Fine–Gray competing risk models (Supplementary Table 8). Strikingly, we observed a consistent and strong protective association of a greater proteomic CRF score for cardiovascular, metabolic and neurological outcomes (but not with most cancers). Moreover, the proteomic CRF score improved risk prediction beyond standard risk factors, with improved discrimination and reclassification across nearly every endpoint (for example, all-cause mortality: *C*-index 0.75 to 0.77, *P* < 0.001; cardiovascular mortality: *C*-index 0.79 to 0.82, *P* < 0.001; Fig. 3a). Reclassification was substantial, with a near 30–40% net reclassification beyond clinical risk factors for most conditions across several systems.

To evaluate whether the strong associations with clinical outcomes were confounded by proteomic markers of disease in the CARDIA cohort from which the proteomic CRF score was derived, we conducted a sensitivity analysis by deriving the proteomic CRF from a subset of the CARDIA study cohort that excluded participants with a history of cardiovascular disease (CVD—myocardial infarction, stroke, heart failure, carotid artery disease, peripheral artery disease), diabetes and hypertension. This proteomic CRF score was then translated for use in the UKB in the same manner, and we observed directionally consistent results as our primary analysis with slightly decreased effect sizes (Supplementary Tables 9–12).

### Integration of a proteomic CRF score and polygenic risk

Previous reports have highlighted the complementary impact of polygenic risk and lifestyle in human disease^31–34^. Given the centrality of CRF as an integrative measure of human health, we next explored interaction between the proteomic CRF score and polygenic risk of common diseases (Fig. 3b and Supplementary Table 13). We constructed models for six conditions with established polygenic risk scores (PRS) within the UKB, as a function of the proteomic CRF score, a corresponding PRS and their multiplicative interaction with adjustments for age, sex, race and four principal components of genetic ancestry. While several PRS-by-proteomic CRF score interactions reached weak statistical significance (including CVD and type 2 diabetes), the effect sizes were marginal. Overall, we observed a substantial and additive effect between the proteomic CRF score and each PRS on the corresponding disease outcome, with highest hazards of disease observed among those participants with the lowest proteomic CRF score (corresponding to poor CRF) and high genetic risk (Fig. 3c). For most conditions, the standardized estimates for the proteomic CRF score were on the order of (or higher than) those for PRS (for example, diabetes: HR_proteome_ = 0.37, 95% CI 0.35–0.40; HR_PRS_ = 1.97, 95% CI 1.83–2.12).

### Association of a parsimonious proteomic CRF score with clinical risk

Even with regularization in regression, one main limitation in most multivariable proteomic approaches is the lack of sufficient reduction in molecular dimension to permit clinical translation^16^ (for example, 307 proteins in our recalibrated proteomic CRF score used in UKB). To address the feasibility of clinical translation, we constructed an ‘abbreviated’ score including coefficients from the top 21 most important proteins (ranked by absolute value of the LASSO beta coefficient). We selected 21 proteins since Olink currently offers 21-plex absolute quantification panels. In CARDIA, this abbreviated 21-protein score was correlated with CRF (*ρ* = 0.71). In UKB, we observed consistent effect sizes for nearly all outcomes between the recalibrated proteomic CRF score (307 proteins) and the abbreviated 21-protein score, albeit with generally slightly lower effect sizes for the abbreviated CRF score (Fig. 3d and Supplementary Table 7). These results support plausibility of translation of these results as a biomarker panel of CRF that can be measured at the scale necessary to offer clinical utility.

### Dynamicity of the proteomic CRF score with training

**Fig. 4:**
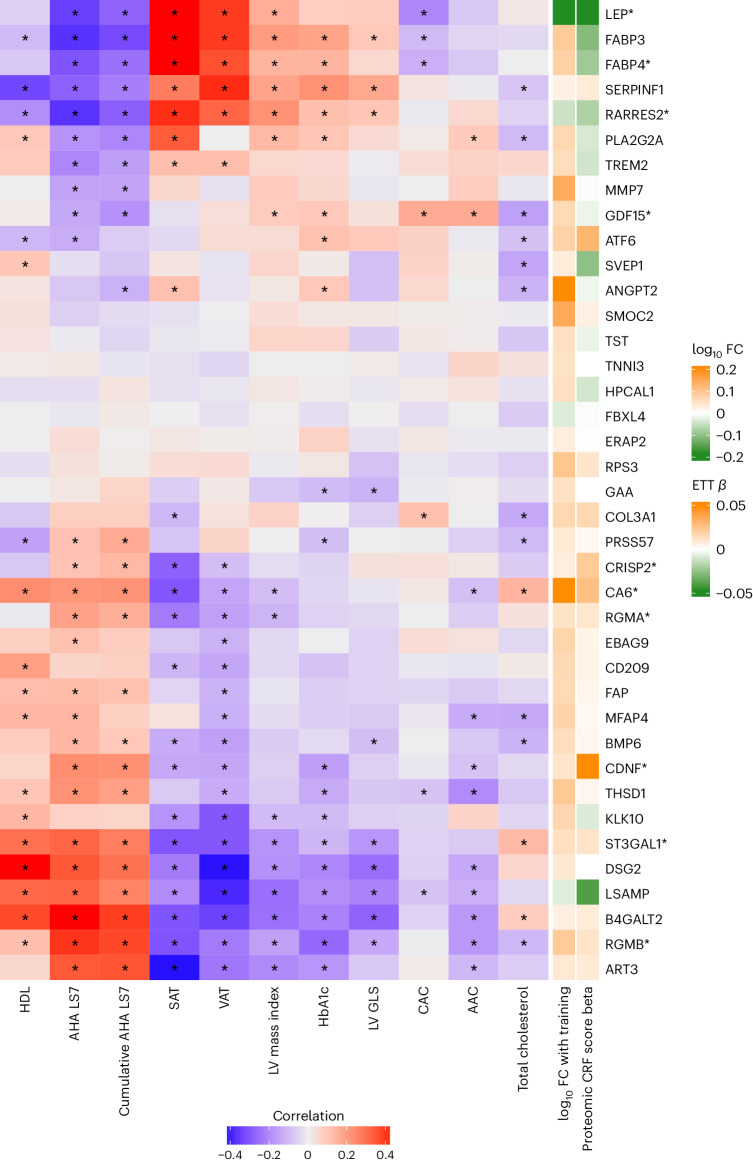
Proteins related to CRF whose levels are dynamic with exercise training are related to cardiometabolic risk factors and diseases. Heatmap of Pearson correlations between individual proteins and cardiometabolic risk factors and disease in CARDIA using the CARDIA validation sample (*N* = 589–669). Proteins visualized are included in the proteomic CRF score and change after a 20-week exercise intervention in HERITAGE (false discovery rate < 5%). Proteins marked with an asterisk are included in the abbreviated 21-protein score. Cells marked with an asterisk indicate Pearson correlations with false discovery rate < 5%. AAC, abdominal aorta calcification; AHA LS7, American Heart Association Life Simple 7; CAC, coronary artery calcification; DBP, diastolic blood pressure; eGFR, estimated glomerular filtration rate; FC, fold change; GLS, global longitudinal strain; HbA1c, hemoglobin A1c; HDL, high density lipoprotein; LV, left ventricular; PA, physical activity; SAT, subcutaneous adipose tissue; SBP, systolic blood pressure; VAT, visceral adipose tissue.

**Extended Data Fig. 3:**
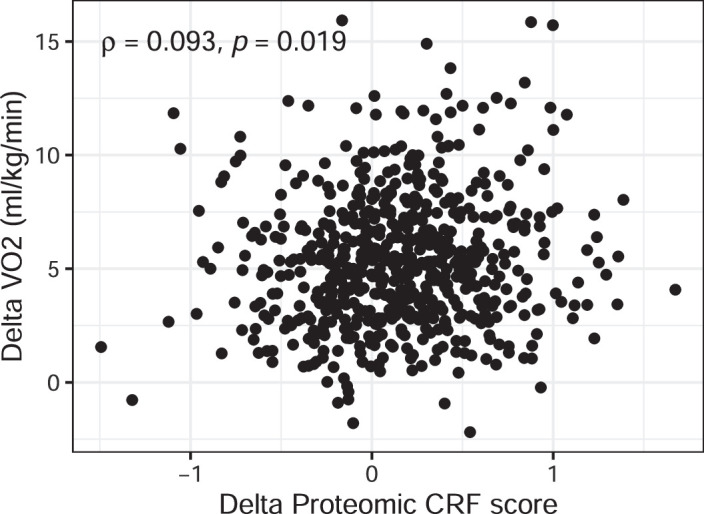
Correlation of change in proteomic CRF score with change in peak VO_2_ with exercise training in HERITAGE. After a 20-week exercise training program in HERITAGE, we observed correlation between changes in the proteomic CRF score with changes in peak VO_2_, which were replicated in regression models. P value is from two sided Spearman rank correlation test.

To leverage the human proteome for CRF assessment, it is critical to evaluate its potential for modification through intervention. After a 20-week exercise training program in HERITAGE^35^, we observed an increase in the recalibrated (nonabbreviated) proteomic CRF score (paired *t*-test, 0.14; 95% CI, 0.11–0.18; *P* = 2.5 × 10^−15^), which was correlated with a change in peak VO_2_ (Extended Data Fig. 3). In regression modeling, we found that a change in the recalibrated proteomic CRF score was associated with a change in peak VO_2_ (1 s.d. increase in recalibrated proteomic CRF score ≈ 0.84 ± 0.25 ml kg^−1^ min^−1^ increase in peak VO_2_; *P* = 8.5 × 10^−4^), independent of age, sex, race, BMI, pretraining peak VO_2_ and pretraining recalibrated proteomic CRF score. There were no differences in the response to changes in the proteomic CRF score with training by sex (*P* = 0.62). Additionally, we examined whether the pretraining proteomic CRF score was associated with the VO_2_ response to training, and observed that a higher recalibrated proteomic CRF score was associated with a greater increase in peak VO_2_ with training, independent of age, sex and race (0.59 ± 0.17 ml kg^−1^ min^−1^ increase per 1 s.d. increase in recalibrated proteomic CRF score; *P* = 6.4 × 10^−4^), with mitigation of the association when further adjusted for BMI (0.30 ± 0.17 ml kg^−1^ min^−1^ increase per 1 s.d. increase in recalibrated proteomic CRF score; *P* = 0.08). Constituents of the proteomic CRF score that exhibited significant changes with 20-week training in HERITAGE^36^ were correlated with an array of metabolic, vascular and myocardial phenotypes in CARDIA (Fig. 4 and Supplementary Table 14). Several of these proteins exhibit clinical and molecular plausibility, with reduction in adiposity (LEP), lipid metabolism (RARRES2), regulation of bone morphogenic protein pathways (RGMB) and mitigation of ischemia-reperfusion injury (CDNF^37^) among others. Many were not related to cardiometabolic phenotypes in CARDIA, suggesting potential new mechanisms of benefit.

## Discussion

The notion that tissue-specific, exercise-responsive biomolecules (‘exerkines’^35,38^) mirror the metabolic benefits of physical exercise has prompted various efforts to catalog these biomolecular changes^8,10,11,13,16,39^. Several studies have highlighted acute metabolic changes during physical exercise that are linked to important physiological processes such as insulin resistance, inflammation and metabolic health across a wide array of mediators (for example, metabolites^8,11,39,40^, proteins^10,16^ and transcripts^11,41^), some of which overlap in association with total habitual physical activity^12^. While all biomolecule types offer relevant insights as functional biomarkers of CRF, the proteome can rapidly capture functional information (a ‘cause’ and ‘effect’ of CRF), broad cellular processes (with direct pathway implication) and application to a clinical setting as a quantifiable blood-based surrogate of CRF.

Here, we studied a diverse group of 14,145 individuals with varied modes of CRF assessment to characterize the circulating proteomic architecture of CRF. Beginning in a sample of 2,238 middle-aged Black and white adults in the CARDIA study, we successfully developed and validated a broad-based proteomic signature of CRF (‘proteomic CRF score’) using symptom-limited treadmill exercise test that displayed a consistent relation across submaximal treadmill exams in 10,320 individuals in the UK (Fenland, estimated maximal VO_2_) and maximal cardiopulmonary exercise tests (CPETs) in 1,587 individuals in the USA (BLSA, treadmill VO_2_; HERITAGE, cycle VO_2_). Proteins included in the proteomic CRF score specified pathways canonically implicated in CRF biology across several systems, including inflammation and hemostasis, muscle and adipose physiology, pathways of energy and fuel metabolism, oxidative stress and neuronal survival, among others. In 21,988 UKB participants, we observed two key findings of clinical relevance. First, the proteomic CRF score was strongly, independently associated with a range of metabolic, cardiovascular and neurological clinical outcomes, many displaying significant prognostic improvement over standard risk factors (via reclassification and discrimination metrics). Second, these associations appeared to be additive to polygenic risk, suggesting a role for multiomic evaluation in clinical risk assessment. These prognostic relations were maintained using an abbreviated 21-protein panel (the largest currently available for direct absolute protein quantification with Olink). The proteomic CRF score was also dynamic with a 20-week exercise training program, and was associated with response to training. To our knowledge, these data provide the largest report to date establishing a biologically plausible, population-based proteomic biomarker of CRF across a diverse setting, linking these measures to phenotypes and precision medicine risk assessment approaches (including human genetics) longitudinally.

Although other studies have demonstrated the ability of broad circulating proteomics to predict diverse health outcomes^16^, the highest priority protein targets are likely to differ for each outcome, presenting challenges for developing unifying lifestyle or pharmacological approaches for broad risk modification or health promotion. In line with established relations of greater CRF itself with protection from a wide array of adverse cardiovascular^2,42^, respiratory^43^, oncological^44^ and neurocognitive outcomes^45^, we observed a proteomic signature trained on CRF (‘proteomic CRF score’) was associated with diverse clinical outcomes in a large sample of around 22,000 UKB participants (an order of magnitude larger than previous studies^16^). Beyond merely establishing a statistical association, the proteomic CRF score offered significant improvement in risk reclassification and discrimination across several conditions (for example, all-cause death, cardiovascular death, diabetes), suggesting its potential to augment clinical risk prediction. Moreover, in line with previous work demonstrating lack of strong interaction between genetics and lifestyle^31^, proteomic and genetic risk were complementary, with the highest clinical risks observed for those individuals with both high proteomic and genomic risk and a lowered risk for those individuals with high proteomic CRF across genetic risk. A critical finding was that these associations were robust to increased parsimony via an abbreviated 21-protein proteomic CRF score, laying groundwork for future studies of clinical translation. In this context, a proteomic CRF score may have clinical utility as a surrogate of CRF to extend its applicability to resource-limited settings, older adults or individuals with contraindications to exercise or musculoskeletal disabilities (with impaired achievement of peak exercise) in whom direct CRF assessment is challenging.

Given modifiability of CRF with lifestyle interventions (for example, physical activity^46^)—a critical test for any precision biomarker of CRF lies in modifiability with training. After a 20-week exercise training program within HERITAGE, we observed a modest but significant relation between changes in the proteomic CRF score with training and the peak VO_2_, with a 1 s.d. increase in proteomic score corresponding to an increase in peak VO_2_ of nearly 1 ml kg^−1^ min^−1^ (approximately 20% of the mean effect of training in HERITAGE). While HERITAGE is a healthy group (and effect sizes in a clinical population probably vary), 1 ml kg^−1^ min^−1^ is considered a ‘clinically actionable’ effect size in CVD^47^: in the HF-ACTION trial, an increase in peak VO_2_ of approximately 0.9 ml kg^−1^ min^−1^ was associated with a ~5% lower risk of mortality^48^. This effect size is greater than the median 3-month increase in peak VO_2_ observed among HF-ACTION participants randomized to exercise intervention (0.6 ml kg^−1^ min^−1^), but is on par with effects of diet and exercise within a trial of participants with HFpEF^49^. Moreover, we observed an association between pretraining proteomic score and changes in peak VO_2_ with training. These findings contribute new contributory evidence on the plasticity of the proteomic CRF biomarker, supporting broad, ongoing efforts to develop multiomic biomarkers of CRF with divergent exercise and training regimens toward personalization of exercise training responses^50^.

The innovation of our approach is contextualized by a rich history of approaches targeting CRF prediction to ease clinical translation. Indeed, previous work to develop nonexercise prediction models of CRF has spanned physical activity questionnaires^51–60^, resting heartrate^53,58,60^, BMI/body composition^51–63^, genetics^64^, proteomics^16^, metabolomics^13^ and activity monitor data^61–63,65^. However, most previous studies have been conducted in healthy or trained individuals and lack a demonstration of strong relations with to multisystem clinical outcomes. The current approach represents a notable advance, merging populations at higher metabolic risk (mirroring the advancing prevalence of cardiometabolic diseases worldwide), modes of exercise, a broad proteomic space, with several validation samples incorporating human genetics (UKB), subclinical phenotypes (CARDIA) and exercise training response (HERITAGE). As precision medicine approaches advance, incorporation of several methods (for example, wearable activity monitor plus ‘omics’) to refine clinically translatable estimates of CRF are likely to improve on any single method.

While biological plausibility and reproducibility of previous smaller studies suggest external validity, several important limitations of this work merit discussions. CRF assessments were not standardized across cohorts, which were themselves variable by age, geography, race and time epoch, although this heterogeneity may also be viewed as a strength since it highlights the robustness of our approach through successful crossvalidation. In addition, there was an interval of around 5 years between the proteomic and CRF assessment in CARDIA, which may have introduced additional variability in our estimates. However, replication of our multivariable proteomic CRF score across three additional studies (Fenland, HERITAGE and BLSA), and demonstration of its modifiability with exercise training (HERITAGE) testifies to the transportability of this approach. Although our study was limited in representation of older adults, the prognostic utility of proteomics independent of age, sex and race are a testament to potential clinical relevance. The proteomic platform utilized in the derivation samples was aptamer-based (SomaScan), which has some limitations in terms of specificity on per-protein level^66^. Nonetheless, we validated the clinical associations of these signatures in a different platform (Olink) in a broader set of individuals (UKB). The assessment of outcomes in UKB was administrative, with potential attendant misclassification and ascertainment biases, which we would anticipate leading to a bias toward null association. Additional forthcoming consortium-level studies across a wider range of exercise types will be important tools to study for potential sex-specific differences and may help clarify proteomic effects from changes in metabolic or lifestyle factors and CRF^50^.

In summary, we define, characterize, and validate a CRF-related proteome across four studies including approximately 14,000 individuals, spanning age, sex, race, geography and type of CRF assessment. CRF-related proteins demonstrated biological plausibility (including consistency with previous studies) and identified individuals with high risk of adverse clinical events across a wide array of organ systems in around 22,000 individuals. Proteomic risk appeared additive to polygenic risk and was maintained down to a clinically actionable proteomic panel. These results suggest the potential for population-based proteomics to provide a biologically relevant, clinically actionable molecular barometer of CRF with clinical potential.

## Methods

### Population-based cohorts

#### Coronary Artery Risk Development in Young Adults

The CARDIA study is a prospective, population-based, cohort study designed to study risk factors for cardiovascular disease development through the lifecourse. The original study commenced in 1985–1986 across four US field centers (Birmingham, AL; Chicago, IL; Minneapolis, MN and Oakland, CA) to study risk factor development throughout young adulthood to midlife, as previously described^72–75^. For this study, we included 2,238 individuals with circulating proteomics (SomaScan) at Year 25 (2010–2011) and ETT time for CRF at year 20 (2005–2006). We intentionally did not refine the CARDIA study population based on reason for stopping ETT or thresholds signifying maximal effort (for example, 85% maximum predicted heartrate) to preserve a maximal sample size and include participants who stopped early for several reasons that may reflect heightened clinical risk. Characterization of demographic, clinical and exercise test data were used as previously published^76,77^. Specifically, CVD was defined as a history of myocardial infarction, heart failure, stroke, carotid artery disease and peripheral artery disease. Participants provided written informed consent and approval to use deidentified data from CARDIA for this study was provided by the Institutional Review Board (IRB) at Vanderbilt University Medical Center (IRB no. 211402).

#### Fenland

The Fenland Study is a population-based cohort study of 12,435 participants (born between 1950 and 1975) recruited from general practices in Cambridgeshire, UK, from January 2005 to April 2015^78^. Exclusion criteria were known diabetes, pregnancy or lactation, inability to walk unaided for a minimum of 10 min, psychosis or terminal illness. Our analytic sample included 5,473 women and 4,847 men with available CRF testing, proteomic and clinical data who attended one of three study sites (Cambridge, Ely or Wisbech). The study was approved by the Cambridge Local Research Ethics Committee (NRES Committee, East of England Cambridge Central, reference no. 04/Q0108/19). All participants provided written informed consent for blood sample measurements, exercise testing and other assessments beyond the baseline examination.

#### Baltimore Longitudinal Study of Aging

The BLSA is a prospective, longitudinal cohort study commenced in 1958 to study age-related conditions^15,79^. Our analytic sample included 845 participants who had undergone CPETs and had circulating plasma proteins quantified at the same time. Demographic and exercise data were defined as previously published^80^. The BLSA study protocol was approved by the Internal Review Board of the Intramural Research Program of the National Institutes of Health (protocol no. 03AG0325) and all participants provided written informed consent at each visit.

#### Health, Risk Factors, Exercise Training and Genetics study

HERITAGE is a study of the genetic and nongenetic contributors to biological responses to aerobic exercise training^81^. Participants were recruited as family units with African or European descent at five centers in the USA and Canada between 1992 and 1997, as described^81^. Participants had to be healthy without cardiometabolic disease but with a sedentary lifestyle for the 3 months preceding enrollment. We included published association data from 742 participants with directly measured maximal aerobic capacity (peak VO_2_) before exercise training and circulating proteomics^10^. Proteomic changes after a 20-week training period were also included^36^. All participants provided written informed consent. The IRB at Beth Israel Deaconess Medical Center approved this study (IRB no. 2016P000186).

#### UK Biobank

The UKB is a population-based study of >500,000 participants aged 40–69 years when recruited between 2006 and 2010 across the UK. UKB was constructed to enable large-scale scientific discoveries of human health^82^. Recently, the study coordinators released proteomics data using the Olink Explore 1536 panel on approximately 52,000 UKB participants. Our analytic sample included 21,988 participants without missing values for the proteins used to calculate a proteomic score of CRF. Approval for UKB access is under proposal no. 57492.

To maximize external validity and generalizability across broad populations, we selected CARDIA as the discovery cohort to develop a proteomic score of CRF, despite 5-year differences between proteomic and CRF assessments. Unlike Fenland and HERITAGE, which excluded participants with prevalent cardiometabolic disease, CARDIA is a population-based study inclusive of prevalent conditions. While BLSA and UKB included participants with prevalent cardiometabolic disease, the number of participants with both CRF and proteomic data is less than half of that in CARDIA. Additional considerations that guided our selection of CARDIA include its broad proteomic coverage (7k SomaScan versus 5k SomaScan in HERITAGE, Fenland and Olink Explore 1536 in UKB), and use of a symptom-limited maximal stress test (Fenland and UKB impute peak VO_2_ data from submaximal tests).

### CRF assessment

CRF was assessed in CARDIA, BLSA, Fenland and HERITAGE according to cohort-specific protocols. In CARDIA, a symptom-limited ETT (modified Balke protocol) was performed as previously described^76,83,84^. Each test consisted of a maximum 18 min, with changes in treadmill speed or grade every 2 min with a maximum workload of 19 metabolic equivalents of task (METs) (for example, 5.6 miles per hour and 25% incline). Participants were excluded from ETT if they had cardiovascular or pulmonary diseases, musculoskeletal diseases worsened by exercise, uncontrolled metabolic or infectious disease, severe rest hypertension (systolic over 200 mmHg or diastolic over 110 mmHg), electrocardiographic features of ischemic heart disease or arrhythmia, pregnancy or at the discretion of exercise personnel. CRF was estimated as the duration of time a participant was able to walk/run on the treadmill. We did not exclude participants based on submaximal or early test conclusion in CARDIA.

In Fenland, CRF was assessed using a submaximal treadmill test (with imputation to maximal effort as described, methods taken from ref.^14^ with attribution provided by this statement) to generate estimated maximal oxygen consumption (peak VO_2_) per kilogram of total body mass. Participants exercised for up to 21 min while treadmill speed and incline increased across four stages. Exercise heartrate response was recorded using a combined heartrate and movement sensor (Actiheart; CamNtech)^85^. The test ended if one of the following criteria were satisfied: (1) levelling-off of heartrate (<3 beats per min (bpm)) despite an increase in workrate; (2) reaching 90% of the participant’s age-predicted maximal heartrate^86^; (3) exercising above 80% of age-predicted maximal heartrate for over 2 min; (4) reaching a respiratory exchange ratio (RER) of 1.1; (5) participant desire to stop; (6) participant indication of angina, light-headedness or nausea; or (7) failure of the testing equipment. Gas exchange measurements were sometimes unavailable for various reasons (for example, participants declining to wear a gas analysis mask, mask fit issues during exercise, system errors) that could be correlated with health-related factors. To mitigate biases that would emerge from the exclusion of participants lacking gas exchange data, and to maintain a standardized approach in estimating peak VO_2_ across the study, we opted to extrapolate the workrate-to-heartrate relationship to age-predicted maximal heartrate. Peak VO_2_ was estimated by extrapolating the linear relationship between heartrate and treadmill workrate^87^ to age-predicted maximal heartrate^86^, adding an estimate of resting energy expenditure, and then converting the resultant workrate value to VO_2_ (ml O_2_ min^−1^ kg^−1^) using a caloric equivalent for oxygen of 20.35 J ml O_2_^−1^.

In HERITAGE, CRF was measured using a cycle ergometer with metabolic cart gas exchange measures with VO_2_ averaged over 20 s intervals, as described^10^. CRF was defined as the peak VO_2_ and exercise peak was determined from at least one of the following: RER >1.1, a plateau in VO_2_ (<100 ml min^−1^ change in the last three measures), or a maximal heartrate within 10 bpm of the age-predicted maximum. After baseline CRF assessment, HERITAGE participants underwent supervised exercise training three times per week for 20 weeks^10^. CRF assessment was then repeated after completion of the training protocol.

In BLSA, CRF was measured using a symptom-limited treadmill exercise test with metabolic cart gas exchange measures using a modified Balke protocol with VO_2_ averaged over 30 s intervals^80^. Exercise testing ended after self-reported exhaustion or health- and/or safety-related stopping criteria occurred. To ensure that the maximal VO_2_ was achieved, the analysis was limited to participants with an RER ≥ 1. Of the 845 participants included in our study, 133 (15%) had RER between 1 and 1.1. Of these participants, 119 (89%) either reached >85% of their age-predicted maximum heartrate (calculated as 220 − age) or rated their exertion during the treadmill test as 17 or great on a 20-point Borg perceived exertion scale.

### Proteomics

Proteomic quantification in CARDIA was performed using aptamer-based technology (Somalogic). Overall, 7,524 circulating aptamers were quantified. A total of 68 participants had more than one measurement of plasma proteins (at the same visit), and their protein data was averaged. We excluded nonhuman proteins (*N* = 233) and proteins with a coefficient of variation >20% (*N* = 61). Using principal component analysis on a matrix of the log-transformed, and scaled proteomic data, we checked visually for batch effects and participant outliers by plotting the first two principal components against each other. No batch effects were detected, and no participant outliers were identified (Supplementary Fig. 1). Fenland (5k aptamer platform), HERITAGE (5k aptamer platform) and BLSA (7k aptamer platform) also used SomaScan proteomics technology with methods described previously^10,16,88,89^. The UKB quantified circulating proteins using the Olink Explore 1536 panel^90^, and we excluded proteins where >40% of measurements were below the limit of detection (*N* = 130) or were missing in >20% of participants (*N* = 3). Of note, as noted above, HERITAGE data was used as published; the remainder of cohorts were analyzed as part of this work.

### Statistical methods

#### Construction and validation of a proteomic score of CRF (‘CRF proteome’)

To explore the multidimensionality of the CRF proteome, we used LASSO regression within a linear modeling framework to develop a multivariable signature of CRF. For the purposes of analysis, the CARDIA cohort was split into a 70% derivation and 30% validation sample balanced on ETT time. The LASSO model was constructed in the CARDIA derivation sample with CRF (ETT time) as the outcome. Adjustments for age, sex, race and BMI were included as unpenalized factors (forced in regression models) with the entire proteome included as penalized factors for selection. Proteins were log-transformed, and proteins and CRF were standardized (mean 0, variance 1) for modeling. Crossvalidation was used for model hyperparameter optimization. Each CARDIA participant’s proteomic CRF score was defined as a linear combination of each protein concentration by the respective model coefficient. We excluded age, sex, race, BMI and intercept coefficients in the score calculation, such that each protein coefficient was conditioned on these covariates (to reduce dependence of the final score on these covariates). Protein scores were standardized (mean 0, variance 1) for downstream analyses.

#### External cohort validation of the CRF proteome

To test the external validity of the CRF proteome across additional cohorts with different proteomic coverages, we employed a recalibration approach. Our recalibration effort used a LASSO model in CARDIA, where the original score (as above) was the dependent variable and all overlapping proteins were included as independent variables. This approach generated coefficients in CARDIA that could be applied to Fenland, HERITAGE and UKB. It was not needed in BLSA, where the platform was the same as CARDIA. Recalibration accuracy (based on correlation between the original score and the recalibrated scores in CARDIA) was excellent (HERITAGE score, Pearson *r* = 0.98; Fenland score, Pearson *r* = 0.99; UKB score, Pearson *r* = 0.93).

#### Relation of the CRF proteome with clinical outcomes and its interaction with polygenic risk

Finally, we performed survival analysis in UKB to estimate the prospective association of the CRF proteome with a broad array of outcomes. Death and death category (cardiovascular death, cancer death, respiratory death) were defined by using death registry data (UKB Data Field 40000) and the International Classification of Disease tenth revision (ICD10) code provided for primary cause of death (UKB Data Field 40001). Mappings for ICD10 data to death category were informed by previous work^91^. The censor dates for death data (and other outcome data) were determined for each participant using the location of initial assessment (UKB Data Field 54) and the region-specific censor dates provided by the UKB. Survival analysis with death outcomes were censored on 30 November 2022 for all alive participants. Survival analysis with incident disease outcomes (for example, chronic obstructive pulmonary disease) were censored on 31 October 2022 for participants in England (*N* = 19,768), 31 July 2021 for participants in Scotland (*N* = 1,356), and 28 February 2018 for participants in Wales (*N* = 864) without events or the death date. Other outcomes in UKB were defined by ICD10 diagnosis codes. To group the ICD10 codes into relevant phenotypes, we used the PheWAS package to generate Phecodes, which represent a composite phenotypes comprised of several related ICD10 codes^92^. For each Phecode, we generated a case, control and excluded status for each participant. Participants with an ‘excluded’ status for a given Phecode were those who had a confounding ICD10 code. This confounding code would not qualify the participant as a case but would disqualify them as being a control. To determine the date of onset for each phenotype, source ICD10 codes were mapped individually to Phecodes, and the date of the earliest qualifying ICD10 code was selected. Prevalent cases were excluded from incident disease models, with prevalent cases being defined as those with a Phecode before their assessment visit, a self-reported diagnosis (UKB Data Field 20002), or a physician diagnosis (UKB Data Fields 2453, 2443, 6150). Details for model phecodes and the corresponding exclusion criteria are listed in the Supplementary Table 7.

Models were constructed using standard Cox regression with the proteomic CRF score as the predictor and the following nested adjustments: (1) unadjusted; (2) age, sex, race; (3) age, sex, race, Townsend deprivation index, body mass index, diabetes, smoking status, alcohol use, systolic blood pressure, low-density lipoprotein (LDL); (4) age, sex, race, Townsend deprivation index, body mass index, diabetes, smoking status, alcohol use, systolic blood pressure, LDL, fat mass as measured by bioimpedance (UKB Data Field 23101). We compared survival models using the maximal set of adjustments with and without the proteomic CRF score to examine differences in C-statistics and net reclassification index (NRI; calculated at the 75th percentile for NRI for events). Our primary analysis for cause-specific death used a ‘cause-specific’ approach where participants without the event of interest (for example, CVD death) are censored at the time of last known vital status or time of death from another cause (for example, cancer death). This approach was complemented using a competing risk framework with a Fine–Gray model with separate models for each of the three modes of death analyzed (for example, CVD, cancer, respiratory). For incident disease models, participants who did not experience the event were censored at the region-specific censor date or the date of death.

To examine potential complementarity of the CRF proteome with polygenic risk of diseases associated with CRF, we used Cox regression models with proteomic CRF score and standard polygenic risk score (UKB Fields 26206, 26212, 26223, 26244, 26248, 26285 (ref. ^93^)) as independent variables (with an interaction term between the two) with adjustments for age, sex, race and four principal components of genetic ancestry (UKB Field 26201).

To examine the potential for clinical translation, we examined performance of a 21-protein score (the maximum number of proteins in an absolute quantification Olink panel currently available) with the recalibrated protein score (307 proteins) in standard Cox models in UKB and compared beta coefficients on the two versions of the CRF proteome. The 21 proteins selected were the top 21 proteins from the recalibrated 307-protein score LASSO model, ranked by the absolute value of the beta coefficients.

#### Dynamicity of CRF proteome with exercise training

Finally, to examine the modifiability of the proteomic CRF score with exercise training and how it tracks with changes in peak VO_2_, in HERITAGE we used paired *t*-tests and regression models for change in peak VO_2_ as a function of change in proteomic CRF score with adjustments for age, sex, race, BMI, pretraining peak VO_2_ and pretraining proteomic CRF score. To test whether the proteomic CRF score was associated with the response to exercise training, we used a model of posttraining peak VO_2_ as a function of pretraining proteomic CRF score adjusted for baseline peak VO_2_, age, sex, race and BMI.

Analyses were conducted with R v.4 or later. All *P* values reported are from two-sided tests.

### Reporting summary

Further information on research design is available in the Nature Portfolio Reporting Summary linked to this article.

## Online content

Any methods, additional references, Nature Portfolio reporting summaries, source data, extended data, supplementary information, acknowledgements, peer review information; details of author contributions and competing interests; and statements of data and code availability are available at 10.1038/s41591-024-03039-x.

### Supplementary information

Supplementary Information Supplemental Fig. 1.

Reporting Summary

Supplemental Tables 1–14 Supplemental Tables 1–14.

## Data Availability

Data for this study are publicly available via the CARDIA coordinating center (www.cardia.dopm.uab.edu), the Fenland Study coordinating center (https://www.mrc-epid.cam.ac.uk/research/data-sharing/), published data from HERITAGE^10,35^ and the UKB (https://www.ukbiobank.ac.uk). Participants did not consent to unrestricted data sharing at the time of study conduct for BLSA. Data from BLSA may be obtained via application to the BLSA coordinating center (https://www.blsa.nih.gov).

## Code availability

Statistical code for the analyses can be found at https://github.com/asperry125/CRF-Proteomics.
