# Supplementary material for: Proteomic analysis of cardiorespiratory fitness for prediction of mortality and multisystem disease risks
Source: Nat Med. 2024 Jun 4;30(6):1711–21. doi: 10.1038/s41591-024-03039-x (PMC11186767; doi:10.1038/s41591-024-03039-x)

nature medicine

Supplementary information https://doi.org/10.1038/s41591-024-03039-x

Proteomic analysis of cardiorespiratory fitness for prediction of mortality and multisystem disease risks

In the format provided by the authors and unedited

Proteomic analysis of cardiorespiratory fitness for prediction of mortality and multi-system disease risks

SUPPLEMENTAL MATERIAL

Supplemental Figure 1: Assessment of batch effect and participant outliers in CARDIA proteomics. CARDIA proteomics were run on 38 plates over a 13 day period. Principal component analysis was used to examine whether there were any batch/plate effects in the CARDIA proteomics dataset as well as examine for any outlier observations.

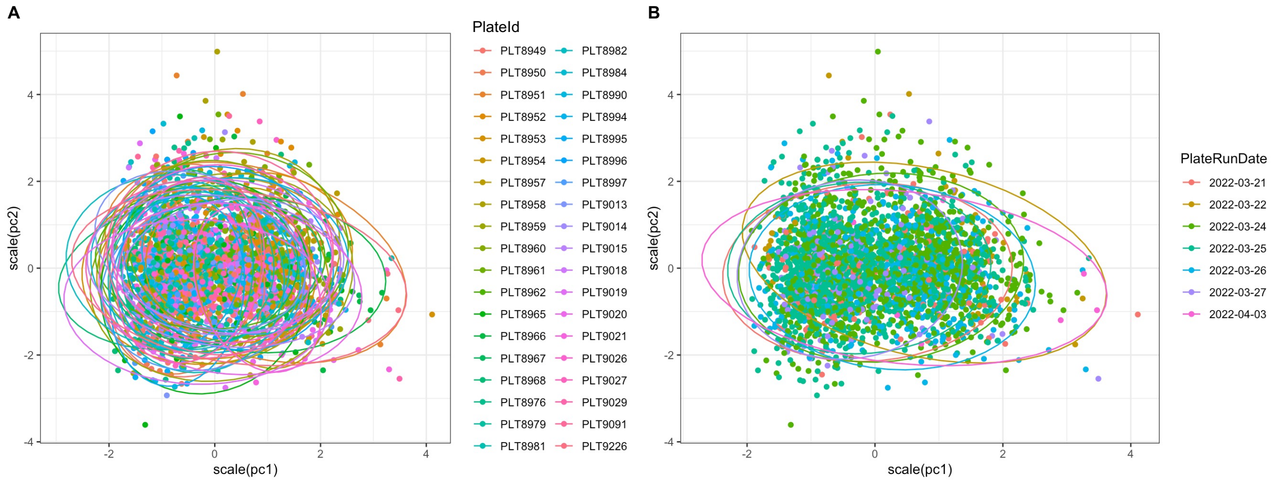

Supplement: Supplementary file 1 — Supplemental Fig. 1. [file 41591_2024_3039_MOESM1_ESM.pdf]
