# Supplementary material for: Proteomic analysis of cardiorespiratory fitness for prediction of mortality and multisystem disease risks
Source: Nat Med. 2024 Jun 4;30(6):1711–21. doi: 10.1038/s41591-024-03039-x (PMC11186767; doi:10.1038/s41591-024-03039-x)
Supplement: Supplementary file 2 — Reporting Summary [file 41591_2024_3039_MOESM2_ESM.pdf]

nature portfolio

Corresponding author(s): Ravi Shah

Last updated by author(s): Apr 16, 2024

Reporting Summary

Nature Portfolio wishes to improve the reproducibility of the work that we publish. This form provides structure for consistency and transparency in reporting. For further information on Nature Portfolio policies, see our Editorial Policies and the Editorial Policy Checklist.

Statistics

For all statistical analyses, confirm that the following items are present in the figure legend, table legend, main text, or Methods section.

n/a	Confirmed	
☐	☒	The exact sample size (*n*) for each experimental group/condition, given as a discrete number and unit of measurement
☐	☒	A statement on whether measurements were taken from distinct samples or whether the same sample was measured repeatedly
☐	☒	The statistical test(s) used AND whether they are one- or two-sided<br>*Only common tests should be described solely by name; describe more complex techniques in the Methods section.*
☐	☒	A description of all covariates tested
☐	☒	A description of any assumptions or corrections, such as tests of normality and adjustment for multiple comparisons
☐	☒	A full description of the statistical parameters including central tendency (e.g. means) or other basic estimates (e.g. regression coefficient) AND variation (e.g. standard deviation) or associated estimates of uncertainty (e.g. confidence intervals)
☐	☒	For null hypothesis testing, the test statistic (e.g. *F*, *t*, *r*) with confidence intervals, effect sizes, degrees of freedom and *P* value noted<br>*Give P values as exact values whenever suitable.*
☒	☐	For Bayesian analysis, information on the choice of priors and Markov chain Monte Carlo settings
☒	☐	For hierarchical and complex designs, identification of the appropriate level for tests and full reporting of outcomes
☐	☒	Estimates of effect sizes (e.g. Cohen's *d*, Pearson's *r*), indicating how they were calculated

Our web collection on statistics for biologists contains articles on many of the points above.

Software and code

Policy information about availability of computer code

Data collection	No software was used.
Data analysis	Statistical code for the analyses can be found at https://github.com/asperry125/CRF-Proteomics. PheWAS package version was 0.99.6-1.

For manuscripts utilizing custom algorithms or software that are central to the research but not yet described in published literature, software must be made available to editors and reviewers. We strongly encourage code deposition in a community repository (e.g. GitHub). See the Nature Portfolio guidelines for submitting code & software for further information.

Data

Policy information about availability of data

All manuscripts must include a data availability statement. This statement should provide the following information, where applicable:

- Accession codes, unique identifiers, or web links for publicly available datasets
- A description of any restrictions on data availability
- For clinical datasets or third party data, please ensure that the statement adheres to our policy

Data for this study are publicly available via the CARDIA coordinating center (www.cardia.dopm.uab.edu), the Fenland study coordinating center (https://www.mrc-epid.cam.ac.uk/research/data-sharing/), published data from HERITAGE, and the UK Biobank (https://www.ukbiobank.ac.uk). Participants did not consent to unrestricted data sharing at the time of study conduct for BLSA. Data from BLSA may be obtained via application to the BLSA coordinating center (https://www.blsa.nih.gov).

Research involving human participants, their data, or biological material

Policy information about studies with human participants or human data. See also policy information about sex, gender (identity/presentation), and sexual orientation and race, ethnicity and racism.

Reporting on sex and gender	Sex was defined as participant self-report of sex assigned at birth. Sex is used as an adjustment for all analyses. We did not consider gender in our analysis as this was not considered to be a meaningful contributor to fitness.
Reporting on race, ethnicity, or other socially relevant groupings	Race/ethnicity was defined by self-report and used as an adjustment in all analyses. As the UK Biobank reports numerous, detailed categories of ethnicity, we grouped ethnicities into broader categories to reduce the degrees of freedom used by ethnicity in those analyses.
Population characteristics	The study samples from CARDIA consisted of 2238 individuals with a median age 51 years (56% female, 43% Black individuals; Table 1). CARDIA participants were generally overweight (median BMI 29 kg/m2) with a modest prevalence of diabetes (14%) and treated hypertension (26%). The median exercise treadmill test duration was 420 seconds.<br><br>The study sample from Fenland consisted of 10320 individuals with a median age of 48 years (53% female, 95% White). The median estimated VO2 max for men was 43 ml/kg/min and for women was 35 ml/kg/min.<br><br>The study sample from HERITAGE consisted of 742 individuals with a median age of 31 years (55% female, 38% Black). The median peak VO2 was 35 ml/kg/min in men and 27ml/kg/min in women.<br><br>The study sample from BLSA consisted of 845 individuals with a median age of 68 years (54% women, 25% Black). The median peak VO2 was 25 ml/kg/min in men and 22 ml/kg/min in women.<br><br>A subsample of the UK Biobank (N=21988; median age 58 years, 54% female, 93% White) with available proteomics was used to test the association of the CRF proteome with a broad array of outcomes.
Recruitment	Participants from all cohorts were recruited from the local communities for each cohort's study center(s).
Ethics oversight	Participants provided written informed consent in each of the respective cohort studies used in this analysis. Approval for this study was provided by the following for analyses within each cohort:<br><br>CARDIA: Institutional Review Board at Vanderbilt University Medical Center (IRB number: 211402)<br><br>Fenland: Cambridge Local Research Ethics Committee (NRES Committee – East of England Cambridge Central, ref. 04/Q0108/19)<br><br>BLSA: Internal Review Board of the Intramural Research Program of the National Institutes of Health (protocol 03AG0325)<br><br>HERITAGE: IRB at Beth Israel Deaconess Medical Center approved this study (IRB number: 2016P000186)<br><br>UK Biobank: Approval for UK Biobank access is under proposal 57492.

Note that full information on the approval of the study protocol must also be provided in the manuscript.

Field-specific reporting

Please select the one below that is the best fit for your research. If you are not sure, read the appropriate sections before making your selection.

☒ Life sciences ☐ Behavioural & social sciences ☐ Ecological, evolutionary & environmental sciences

For a reference copy of the document with all sections, see nature.com/documents/nr-reporting-summary-flat.pdf

Life sciences study design

All studies must disclose on these points even when the disclosure is negative.

Sample size	A prior report used elastic net regression to create a protein score of cardiorespiratory fitness in the HERITAGE cohort. In that study, the investigators had 585 participants, with >5000 proteins assayed. They reported an R squared for model fit of 0.80 and 0.72 in their derivation and validation models, respectively. We used all available samples in CARDIA, N=2238, and based on the prior analyses performed in HERITAGE we felt this sample size was adequate for our study.
Data exclusions	We excluded participants without complete data on model predictors, outcomes or covariates.
Replication	Our study uses a discovery cohort (CARDIA) with 3 replication cohorts (Fenland, HERITAGE, BLSA) to ensure reproducibility of our findings.
Randomization	As our study was designed to develop and validate a biomarker panel of cardiorespiratory fitness and test its association with clinical outcomes, randomization was not necessary.

Blinding	As randomization was not performed, blinding was not necessary.

Reporting for specific materials, systems and methods

We require information from authors about some types of materials, experimental systems and methods used in many studies. Here, indicate whether each material, system or method listed is relevant to your study. If you are not sure if a list item applies to your research, read the appropriate section before selecting a response.

Materials & experimental systems

n/a	Involved in the study
☒	☐ Antibodies
☒	☐ Eukaryotic cell lines
☒	☐ Palaeontology and archaeology
☒	☐ Animals and other organisms
☒	☐ Clinical data
☒	☐ Dual use research of concern
☒	☐ Plants

Methods

n/a	Involved in the study
☒	☐ ChIP-seq
☒	☐ Flow cytometry
☒	☐ MRI-based neuroimaging

Plants

Seed stocks	N/A
Novel plant genotypes	N/A
Authentication	N/A
